# Kidney failure in the course of focal segmental glomerulonephritis in a patient after alloHSCT - a case study and review of the literature

**DOI:** 10.1007/s40620-025-02375-6

**Published:** 2025-10-07

**Authors:** Aleksandra Kaszyńska, Małgorzata Kępska-Dzilińska, Ewa Karakulska-Prystupiuk, Agnieszka Perkowska-Ptasińska, Jolanta Małyszko

**Affiliations:** 1 Department of Nephrology, Dialysis and Internal Medicine, Medical University of Warsaw, Banacha 1A, 02-097 Warsaw, Poland; 2 Department of Hematology, Transplantation and Internal Medicine, Medical University of Warsaw, Banacha 1A, 02-097 Warsaw, Poland; 3 Department of Pathology, Medical University of Warsaw, Pawinskiego 3, 02-097 Warsaw, Poland

**Keywords:** Allogenic stem cell transplantation, Graft versus host disease, Nephrotic syndrome, Focal segmental glomerulosclerosis, Ruxolitinib

## Abstract

Nephrotic syndrome is a rare complication of allogeneic haematopoietic stem cell transplantation (alloHSCT). Its pathogenesis is not fully understood. Membranous nephropathy and minimal change disease are the most common causes of nephrotic syndrome. Nephrotic syndrome occurs more frequently in people with chronic graft-versus-host disease (GvHD). Glucocorticosteroids and cyclosporine are the most common treatments, but their use is associated with the risk of side effects and variable responses. We present a case of a patient after alloHSCT with advanced GvHD who developed nephrotic syndrome resistant to immunosuppressive treatment. Additionally, when her kidney function deteriorated, she required kidney replacement therapy. We would like to emphasise the importance of kidney biopsy as a tool for determining the extent of kidney damage and the possible response to therapy.

**Graphical abstract:**

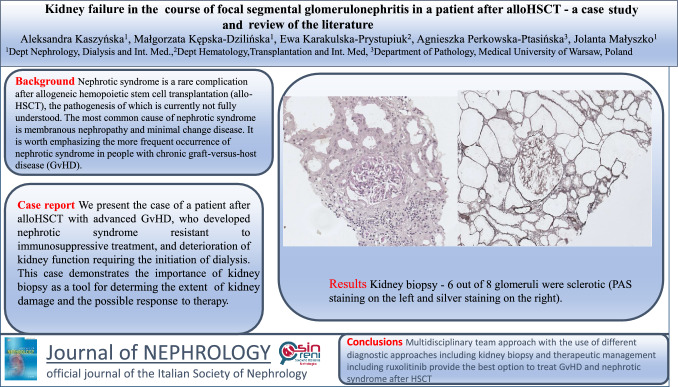

## Introduction

Haematopoietic stem cell transplantation (HSCT) is the most effective treatment for patients with various haematological diseases. However, it may lead to several complications, including kidney disease [1]. Renal dysfunction, including acute kidney injury, chronic kidney disease (CKD), and nephrotic syndrome, may complicate allogenic HSCT (alloHSCT), and is associated with high morbidity and mortality [2]. Management of these renal complications is crucial as they can significantly impact the overall prognosis and quality of life of patients undergoing HSCT. Early identification and intervention are essential to mitigate the risks associated with renal impairment and optimise therapeutic outcomes. Optimising therapeutic outcomes requires a multidisciplinary approach, involving nephrologists, oncologists, and transplant specialists, to ensure comprehensive care. Regular monitoring of kidney function, alongside tailored interventions, can help address the complications associated with kidney diseases in these patients. This collaborative effort not only enhances patient safety but also contributes to improved long-term survival rates and overall well-being. Furthermore, education and support for patients and their families regarding renal health can empower them to play an active role in managing their condition throughout the treatment process. By fostering a strong partnership between healthcare providers and patients, individuals can better understand their treatment options and make informed decisions. This proactive approach not only facilitates adherence to prescribed therapies but also cultivates a sense of community and support that is vital for navigating the challenges of kidney disease. Nephrotic syndrome after alloHSCT is rare, usually diagnosed 6–12 months post-transplant, with membranous nephropathy being the dominant manifestation [3]. The aetiology and pathogenesis of nephrotic syndrome are unclear. However, nephrotic syndrome can be associated with graft-versus-host disease (GvHD) and/or with conditioning regimens consisting of irradiation before HSCT [3]. Data on kidney pathology following HSCT come primarily from the paediatric population and are very limited. Below, we present the clinical case of a patient who underwent alloHSCT for acute myeloid leukaemia, later developing secondary nephrotic syndrome and advanced kidney failure requiring kidney replacement therapy in the form of haemodialysis. This case highlights the potential renal complications associated with HSCT, emphasising the need for careful monitoring and management of kidney function in these patients. Further research is essential to elucidate the underlying mechanisms and improve outcomes in individuals experiencing similar complications.

## Case report

**Fig. 1:**
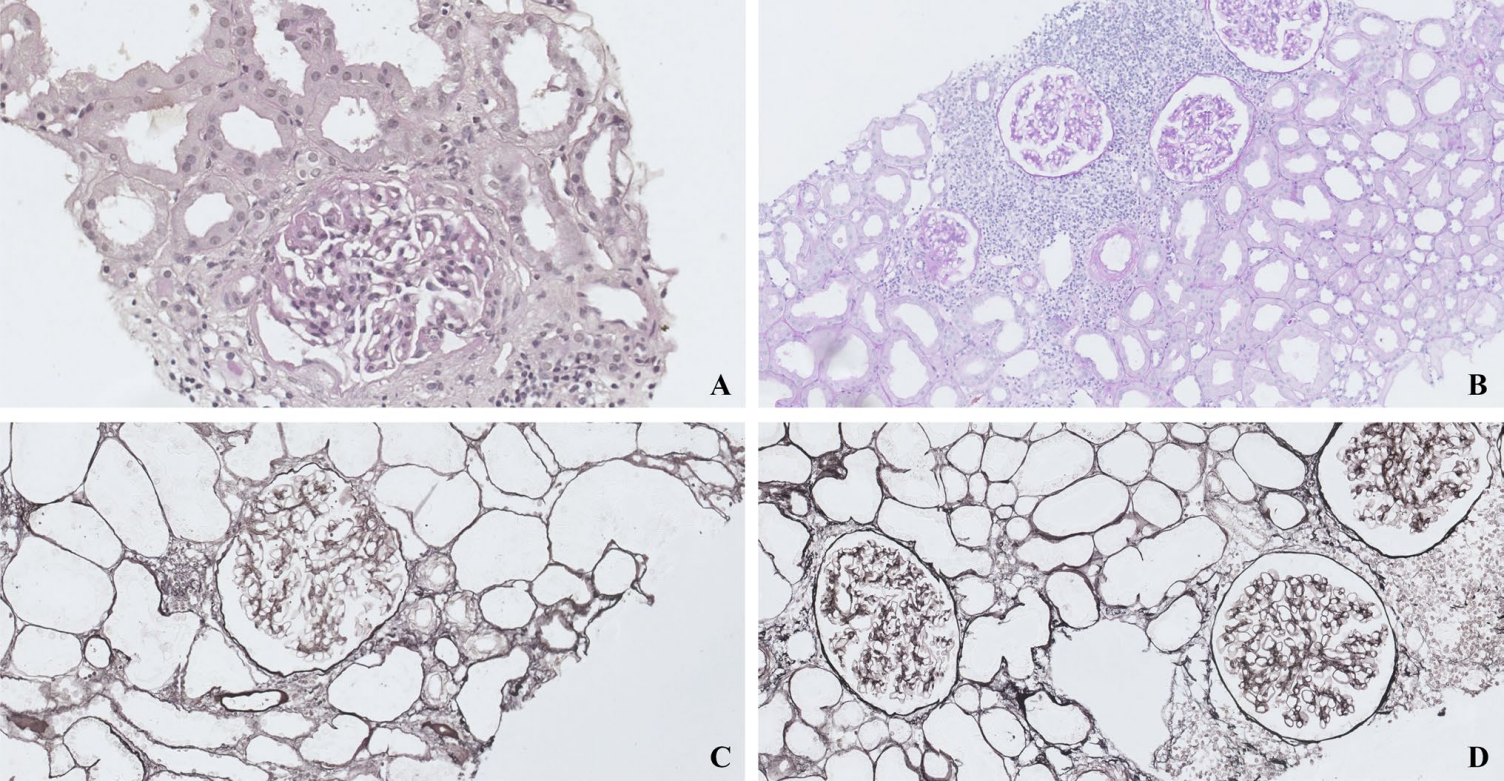
Kidney biopsy with both Periodic acid–Schiff** (**PAS) staining and silver staining with full description of the biopsy findings in the text. **A** PAS staining borderline changes, preserved glomerular structure. **B** silver staining borderline changes, preserved glomerular structure. **C** PAS staining segmental glomerular sclerosis. D silver staining segmental glomerular sclerosis

**Fig. 2:**
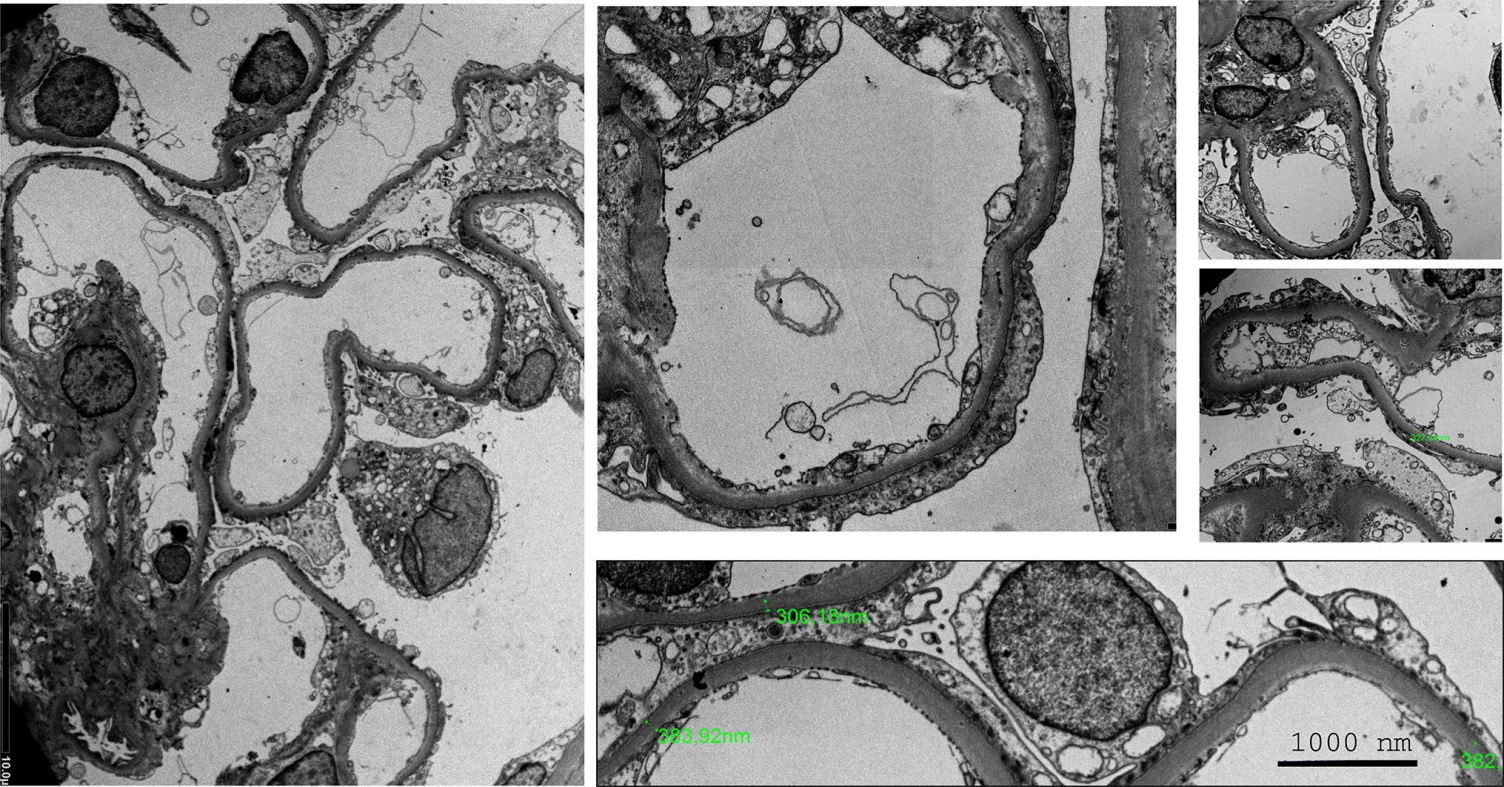
Electron microscopy examination based on sections through two glomeruli with full description of the biopsy findings in the text (calibre of capillary loops given in nm)

A 31-year-old woman who underwent alloHSCT in 2010 was admitted to the Department of Nephrology, Dialysis and Internal Diseases in March 2021 due to increasing proteinuria and worsening kidney function. A significant increase in proteinuria had been observed in the 3 months prior to hospitalisation. In 2010, she was diagnosed with acute myeloid leukaemia. She underwent an allogeneic bone marrow transplantation from her brother. After the alloHSCT, the patient developed severe GvHD, with eye, lung and joint involvement. Nephrotic-range proteinuria had been observed since 2011. The patient was under outpatient haematology care from 2011. She was not referred for a nephrology consult despite onset of proteinuria. Later she declined a nephrology work-up when proposed. Nephroprotection with ramipril was introduced by a haematologist, and proteinuria slightly declined. In March 2021, creatinine concentration was 1.4–1.6 mg/dL, albumin was 2.5 g/dL, and total protein was 5 g/dL. At the same time, immunological testing revealed negative antinuclear antibodies, antineutrophil cytoplasmic antibodies, antibodies against double-stranded DNA, and C3 and C4 complement components within the normal range. Protein excretion in a 24-h urine collection exceeded 12 g. Genetic tests were not performed due to a history of chronic GvHD and the negative family history for kidney disease. Due to heavy proteinuria and elevated serum creatinine (1.4–1.6 mg/dL), she was referred to our department for further work-up. A kidney biopsy performed on March 24th, 2021, was complicated by a haematoma. The biopsy sample size consisted of eight glomeruli. The results of the biopsy were as follows: six out of eight glomeruli in the biopsy were sclerotic, the interstitium revealed moderately diffuse infiltration and moderately diffuse fibrosis, tubules showed moderately diffuse tubular atrophy, arterioles had moderate arterial wall hyalinosis, staining for amyloid was negative. Immunofluorescence showed only sclerotic glomeruli, and staining for IgA, IgM, IgG, C1q, C3, κ, λlight chains and fibrinogen was negative. The conclusion drawn from light microscopy was that due to 6 of 8 glomeruli being sclerotic, and immunofluorescence not adding new data to the diagnosis because of a lack of non-sclerotic glomeruli, the biopsy was considered to be of very limited value. Kidney biopsy with both periodic acid–Schiff staining and silver staining are presented in Fig. 1. Electron microscopy revealed early-stage segmental glomerulosclerosis and significant podocyte damage (podocytopathy) based on only two glomeruli (Fig. 2). The full description of the electron microscopy examination based on sections through two glomeruli is as follows: Capillary loops were of wide calibre. Basement membranes of the capillary loops were of normal thickness. There was a slight increase in mesangial cells and matrix. Podocytes were less numerous, and their foot processes were flattened along most of the basement membranes, with segmental preservation.

The patient later declined a repeat biopsy. Based on the clinical picture and kidney biopsy findings, the nephropathologist’s report stated that there was a high probability of focal segmental glomerulosclerosis (FSGS).

In May 2021, prednisone (1 mg/kg) and ruxolitinib were initiated. Ruxolitinib was added by a haematology specialist as the nephrotic syndrome and FSGS were believed to be secondary to chronic GvHD.

Initially, a promising response was observed with a slight reduction in proteinuria, as well as a decrease in creatinine levels, and amelioration of peripheral oedema. However, when we tried to taper the prednisone dose, both proteinuria and creatinine levels increased. Mycophenolate mofetil (MMF) was then added without significant improvement in proteinuria, followed by a switch to cyclosporine A (CsA), with likewise no effect on proteinuria. Additionally, due to hypogammaglobulinaemia, she continued regular immunoglobulin infusions and erythropoietin-stimulating agents **(**ESAs) were administered for the treatment of severe anaemia. Over the following 6 months, till December 2021, a gradual rise in serum creatinine was observed while proteinuria increased gradually and exceeded 20 g in the 24-h urine collection. Exacerbation of oedema required loop diuretic up-titration. She was admitted to our department in August 2022 with the following data creatinine, 4.4 mg/dL; urea, 74 mg/dL; pH, 7.28; HCO_3_, 17 mmol/L; K, 4.89 mmol/L; haemoglobin, 6.8 g/dL (despite ESA therapy); albumin, 1.9 g/dL; total protein, 4.4 g/dL; and spot proteinuria, 1527 mg/dL. Physical examination revealed massive oedema in the lower limbs, signs of iatrogenic Cushing's syndrome, and numerous wheezes over the lung fields. Diuretic therapy was intensified (intravenous administration) and packed red blood cells were transfused. Due to the lack of improvement, persistent symptoms of overhydration, and worsening kidney function, kidney replacement therapy was indicated. The first haemodialysis (HD) was performed on 30th August, 2022 after the implantation of a tunnelled cuffed catheter. She started regular dialysis three times a week, resulting in a significant improvement in clinical status, disappearance of shortness of breath, and reduction of oedema. Haemoglobin increased to 10–11 g/dL. Ruxolitinib was discontinued following a haematology consult, and steroids were gradually tapered. The most likely cause of the patient's nephrotic syndrome and progressive kidney failure was chronic GvHD.

In January 2023, the patient underwent evaluation for kidney transplantation. The possibility of a living-related transplantation from her brother (stem cell donor) was discussed. Finally, kidney transplantation from a cadaveric donor was performed on 30th August, 2023.

## Discussion

Nephrotic syndrome is a rare complication of bone marrow transplantation but should always be considered, especially when peripheral oedema increases and kidney function deteriorates. Nephrotic syndrome is typically diagnosed approximately 6–12 months after alloHSCT. The incidence in adults is estimated to range from 0.37 to 6.1% [3]. In patients undergoing allogeneic bone marrow transplantation, the most common pathologies causing nephrotic syndrome are membranous nephropathy (MN) and minimal change disease (MCD) [2]. To confirm nephropathy, a kidney biopsy is usually necessary. In a study by Luo et al. [3], nephrotic syndrome was found in 9 of 257 patients who underwent alloHSCT. The 5-year incidence was 3.5%, with average onset occurring 225 days after transplantation. Among patients treated for chronic GvHD, nephrotic syndrome was diagnosed in 7 patients more than 3 months after alloHSCT [3]. In a recent study by John et al. [4], kidney biopsy was performed on 19 allogenic and 5 autologous HSCT recipients out of 2930 patients who underwent HSCT at the Department of Nephrology and Haematology at Christian Medical College, Vellore, South India, between 2005 and 2020. Among allogenic recipients, two clinico pathological patterns were found: thrombotic microangiopathy (TMA, 12/19 [63%]) and nephrotic syndrome, 7/19 [37%]. Of the 7 patients with nephrotic syndrome, MN was observed in 4 (57%) and MCD in 3 (43%). These patients were primarily treated with steroids, while MMF was used as second-line therapy. The authors noted that kidney biopsy was usually performed in HSCT patients with a strong suspicion of renal GvHD, such as concomitant GvHD at other sites, renal dysfunction, or proteinuria occurring after the discontinuation of GvHD prophylaxis.

In another study involving 1198 children after HSCT for either malignant or non-malignant conditions, 25 children underwent kidney biopsy [5]. The main pathology findings were mesangial proliferative glomerulonephritis (MPGN) and FSGS. Only three children had nephrotic-range proteinuria. Four children in this cohort had kidney injury prior to HSCT and their renal pathology showed FSGS and tubulointerstitial nephritis. After alloHSCT, 13 of these 25 paediatric patients exhibited evidence of GvHD, including 9 with acute GvHD and 4 with chronic GvHD, mostly involving the intestine, skin, and/or liver. In the only case of FSGS following HSCT for severe aplastic anaemia, no acute or chronic GvHD was observed [6].

A published case report discussed FSGS as a complication of GvHD in a 54-year-old man with multiple myeloma undergoing HSCT from a human leukocyte antigen-identical sister [7]. In a more recent study, Yap et al. [8] analysed clinical and histopathological data from patients who developed de novo glomerular diseases after HSCT, with 31 of 2222 patients (1.4%) showing de novo glomerular diseases after a mean duration of 2.8 ± 2.7 years post-HSCT. Histopathological diagnoses included TMA (38.7%), MN (25.8%), MPGN (12.9%), MCD (9.7%), FSGS (9.7%), and membranoproliferative glomerulonephritis (3.2%). Beyar-Katz et al. [9] analysed 116 cases of nephrotic syndrome diagnosed post-HSCT performed between 1988 and 2015, and found that the onset of nephrotic syndrome was associated with acute or chronic GvHD in 87.2% of cases. MN was the most frequent pathology (65.5%), followed by MCD (19%).

Glomerular diseases are an important complication in patients undergoing HSCT, affecting approximately 1–2% of all HSCT recipients, reaching up to 700–1400 cases per year worldwide. GvHD is currently considered the dominant aetiological factor for nephrotic syndrome after HSCT [10]. There is likely a causal relationship between cytomegalovirus infection, exposure to radiations, and the occurrence of haemolytic uraemic syndrome. It should be emphasised that nephrotic syndrome may be one of the symptoms of chronic GvHD and is usually diagnosed after discontinuing or reducing the dose of immunosuppressive drugs [10]. The occurrence of nephrotic syndrome is often accompanied by the recurrence of chronic GvHD. Furthermore, in a recent study, Beshensky et al. [11] suggested that kidney dysfunction in the context of less severe chronic GvHD may be unrelated to chronic GvHD and, potentially, is a consequence of drug-related toxicities. As the development of CKD in HSCT recipients is often multifactorial, a kidney biopsy is required to identify the underlying disease aetiology and pathology [12]. However, data on the kidney biopsies of patients with proteinuria after HSCT are extremely limited. Currently, no guidelines exist for the treatment of nephrotic syndrome after HSCT, but systemic treatment with corticosteroids and CsA is most widely used. The decision to initiate immunosuppressive treatment should be based on the patient's risk factors, together with the severity of proteinuria and kidney function [13].

Early trials leading to ruxolitinib approval did not report nephrotoxicity; however, a recent trial in patients with steroid-refractory atypical GvHD reported that 18% of participants experienced elevated serum creatinine and 4% of patients experienced a severe adverse event due to acute kidney injury (AKI) [A Study of Ruxolitinib in Combination With Corticosteroids for the Treatment of Steroid-Refractory Acute Graft-Versus-Host Disease (REACH-1) [Internet]. 2016. https://clinicaltrials.gov/ct2/show/results/NCT02953678. Accessed June 27th, 2025]. Strohbehn et al. [14] identified and reviewed all patients with a prescription for ruxolitinib between 2012 and 2019 (*N* = 414); the most common indication for ruxolitinib was atypical GvHD (47%). They found that moderate-to-severe AKI occurred within 90 days of starting ruxolitinib in 30 (10%) patients, 25 (83%) of whom were being treated for acute GvHD. However, no biopsy was performed. Recently, Ai et al. [15] reported a case of a person who developed post-HSCT nephrotic syndrome and portal hypertensive ascites. A kidney biopsy revealed membranous nephropathy and renal thrombotic microangiopathy with glomerular immune deposits, suggesting antibody-mediated kidney injury. Treatment with ruxolitinib resulted in remission of both nephrotic syndrome and ascites, suggesting a role for cytokines in the pathogenesis. In our case, after the initial success following ruxolitinib introduction, worsening kidney function and an increase in proteinuria were observed. Thus, it appears that ruxolitinib was a kind of ‘rescue’ therapy, albeit initiated too late to improve patient outcome.

**Fig. 3:**
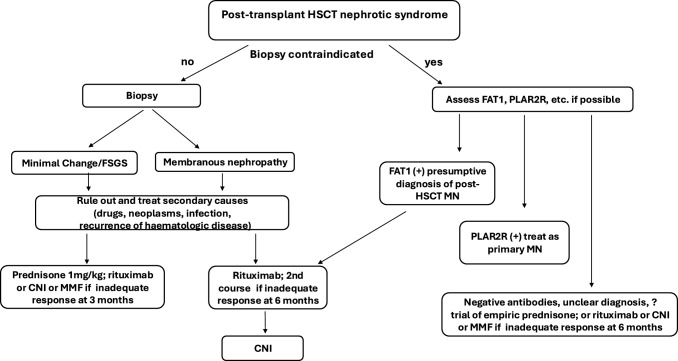
Flow chart with management suggestions in post HSCT nephrotic syndrome. *HSCT* haematopoietic stem cell transplantation, *PLAR2R* phospholipase 2 receptor, *FAT1* FAT Atypical Cadherin 1(protoadherin), *MN* membranous nephropathy, *CNI* calcineurin inhibitors, *MMF* mycophenolate mofetil, FSGS- focal segmental glomerulosclerosis

This case illustrates the importance of a timely kidney biopsy as a guide for determining the extent of kidney damage and aiding in the decision-making process regarding therapy. As proteinuria increased despite nephroprotection, together with a rise in serum creatinine, a kidney biopsy was proposed and performed and it became clear that nephroprotection and preparation for kidney replacement therapy remained the optimal strategies. In our case, the kidney biopsy was performed too late, although at the earliest possible time upon referral to our department. As the person was young and in complete remission from haematological disease, kidney transplantation was considered and discussed. Therefore, the results of kidney biopsy are of additional value in cases of graft glomerulopathy to differentiate between recurrent and de novo changes. We would also like to stress the importance of a multidisciplinary team approach with the use of various therapeutic agents, including ruxolitinib, to manage GvHD and nephrotic syndrome. We have also included a proposed flow chart with management suggestions in post HSCT nephrotic syndrome (Fig. 3).

## Data Availability

The data that support the findings of this study are not publicly available due to containing information that could compromise the privacy of research participants but are available from the corresponding author.
